# Molecular Profiling of Merkel Cell Polyomavirus-Associated Merkel Cell Carcinoma and Cutaneous Melanoma

**DOI:** 10.3390/diagnostics11020212

**Published:** 2021-02-01

**Authors:** Attila Mokánszki, Gábor Méhes, Szilvia Lilla Csoma, Sándor Kollár, Yi-Che Chang Chien

**Affiliations:** 1 Department of Pathology, Faculty of Medicine, University of Debrecen, H-4032 Debrecen, Hungary; gabor.mehes@med.unideb.hu (G.M.); csoma.szilvia@med.unideb.hu (S.L.C.); dr.changchien.yiche@med.unideb.hu (Y.-C.C.C.); 2 Department of Pathology, Kenézy Gyula Teaching Hospital, University of Debrecen, H-4032 Debrecen, Hungary; dr.kollar.sandor@kenezy.unideb.hu

**Keywords:** Merkel cell carcinoma, Merkel cell polyomavirus, melanoma, molecular genetics, next-generation sequencing (NGS)

## Abstract

Merkel cell carcinoma (MCC) is a rare, high-grade, aggressive cutaneous neuroendocrine malignancy most commonly associated with sun-exposed areas of older individuals. A relatively newly identified human virus, the Merkel cell polyomavirus (MCPyV) has been implicated in the pathogenesis of MCC. Our study aimed to examine nine MCC cases and randomly selected 60 melanoma cases to identify MCPyV status and to elucidate genetic differences between virus-positive and -negative cases. Altogether, seven MCPyV-positive MCC samples and four melanoma samples were analyzed. In MCPyV-positive MCC *RB1*, *TP53*, *FBXW7*, *CTNNB1*, and *HNF1A* pathogenic variants were identified, while in virus-negative cases only benign variants were found. In MCPyV-positive melanoma cases, besides *BRAF* mutations the following genes were also affected: *PIK3CA*, *STK11*, *CDKN2A*, *SMAD4*, and *APC*. In contrast to studies found in the literature, a higher tumor burden was detected in virus-associated MCC compared to MCPyV-negative cases. No association was identified between virus infection and tumor burden in melanoma samples. We concluded that analyzing the key morphologic and immunohistological features of MCC is critical to avoid confusion with other cutaneous malignancies. Molecular genetic investigations such as next-generation sequencing (NGS) enable molecular stratification, which may have future clinical impact.

## 1. Introduction

Merkel cell carcinoma (MCC) is a rare, high-grade, aggressive primary cutaneous neuroendocrine malignancy causing rapidly enlarging lesions on sun-exposed areas of older Caucasian individuals [1,2]. The incidence of MCC has increased over the past 30 years due to the aging population, the additional reporting, and improvements in diagnostic techniques [3]. The incidence ranges from 0.1 to 1.6 cases per 100,000 people per year [4,5]. The rising incidence with its often rapidly aggressive course underscores a critical need to analyze the histopathologic and the immunohistochemical (IHC) features of the disease. MCC was regarded as a dermal malignancy that often involves the subcutis. The tumor cells of MCC show a characteristic neuroendocrine cytomorphology with scant cytoplasm and uniform round to oval nuclei., show in the majority of cases, positivity for cytokeratin 20 (CK20), synaptophysin, chromogranin A (CHGA), and neurofilament (NF), but often negative for thyroid transcription factor 1 (TTF-1) [6].

A relatively newly identified human virus, the Merkel cell polyomavirus (MCPyV), has been implicated in the pathogenesis of MCCs [7]. The virus appears to be causative in most cases and it was applied as a diagnostic and prognostic marker [6]. MCPyV is present in around 60–80% of MCC and only in 11% of control tissues within their majority lower copy numbers [8]. Some studies published molecular aberrations that can play a role in the virus-associated MCC malignancy such as *KIT*, *PIK3CA*, and genes in the Hedgehog signal transduction pathway [9,10,11]. In other forms of MCC in which MCPyV cannot be detected, supposing the ultraviolet light exposure represents key drivers in the carcinogenesis [6]. MCPyV-negative MCCs are described by a higher mutation burden compared to MCPyV-positive MCC, and these mutations can be considered UV-signature variations. The most relevant alterations in virus-negative cases affect *NOTCH*, *RB1*, and *TP53* genes [12,13]. In geographic regions with less UV exposure, MCC is more common with MCPyV positivity, whereas in areas with relatively high UV exposure, MCPyV-negative MCCs predominate [14].

Due to limited data available for molecular study in MCCs, particularly with next-generation sequencing (NGS) methods, the aim of our study was (1) to detect the MCPyV status of nine MCC patients, diagnosed from three separate institutes, (2) to compare the molecular differences between virus-positive and -negative subgroups, (3) to identify MCPyV frequency in other randomly selected cutaneous melanoma, and (4) to elucidate genetic differences between virus-associated and -negative cases and MCPyV-associated melanoma samples as well. For this purpose, histology examination, diagnostic IHC including antibody against large T antigen of MCPyV, MCPyV-specific PCR, and NGS with solid tumor gene panel analysis were performed using MCC and MCPyV-associated melanoma samples. Additionally, reverse-hybridization StripAssay was carried out on melanoma samples detecting *BRAF* mutation.

## 2. Materials and Methods

### 2.1. Patients’ Samples

Altogether, nine formaldehyde-fixed paraffin-embedded tissue (FFPE) MCCs and 60 control melanoma samples were tested. All protocols were approved by the authors’ respective Institutional Review Board for human subjects (IRB reference number: 60355/2016/EKU).

### 2.2. Histology and Immunohistochemistry

Hematoxylin and eosin (H&E)-stained slides were carefully analyzed and validated by pathology specialists and appropriate tumor samples were selected for DNA isolation with a tumor percentage >20%. IHC of CK20 (clone Ks20.8, 1:200 dilution, Leica Biosystems, Wetzlar, Germany), synaptophysin (clone 27G12, 1:100 dilution, Leica Biosystems, Wetzlar, Germany), CHGA (clone DAK-A3, 1:400 dilution, Dako, Agilent Technologies, Santa Clara, CA, USA), and TTF-1 (clone SPT24, 1:100 dilution, BioCare Medical, Pacheco, CA, USA) was performed to confirm MCC diagnosis. For the differential diagnosis in melanoma cases, S100 protein (polyclonal, 1:1000 dilution, Leica Biosystems, Wetzlar, Germany), vimentin (clone V9, 1:200 dilution, Leica Biosystems, Wetzlar, Germany), HMB45 (Human Melanoma Black, clone HMB-45, 1:200 dilution, Dako, Agilent Technologies Company, Santa Clara, CA, USA), and Melan-A (clone A103, 1:200 dilution, Dako, Agilent Technologies Company, Santa Clara, CA, USA) antibody was used. MCPyV immunological detection was carried out using MCPyV large T-antigen antibody (clone CM2B4, 1:100 dilution, Merck Millipore, Burlington, MA, USA). Additional staining for Ki-67 (clone MIB1, 1:200 dilution, Dako, Agilent Technologies Company, Santa Clara, CA, USA) was done to determine the cell proliferation index. The p53 antibody (clone Do-07, 1:700 dilution, Dako, Agilent Technologies Company, Santa Clara, CA, USA) was applied to compare immune positivity with molecular genetic findings in MCC cases.

### 2.3. DNA Isolation

Genomic DNA was extracted from FFPE tissues using the QIAamp DNA FFPE Tissue Kit (Qiagen, Hilden, Germany). The isolations were carried out according to the manufacturer’s standard protocol and the DNA was eluted in 50 µL elution buffer. The DNA concentration was measured in the Qubit dsDNA HS Assay Kit using a Qubit 4.0 Fluorometer (Thermo Fisher Scientific, Waltham, MA, USA).

### 2.4. Merkel Cell Polyomavirus Molecular Detection

For virus T antigen and/or viral capsid DNA sequences in the nine MCC and 60 histopathological similar cutaneous malignant melanoma samples, LT1 (large T1), LT3 (large T3), and VP1 (viral capsid protein 1) primer pairs were used. The DNA amplification was carried out according to the earlier study [15]. To demonstrate that the quality and quantity of the DNA samples were acceptable, the human β-globin gene was also amplified.

### 2.5. BRAF StripAssay

Reverse hybridization was carried out using *BRAF* 600/601 StripAssay according to the manufacturer’s protocol (ViennaLab Diagnostics, Vienna, Austria). The assay covers nine clinically relevant mutations in the *BRAF* gene and is certified for human in vitro diagnostics (IVD). For interpretation, hybridization strips were aligned using the standardized layout supplied with the reagents, and positive bands were identified.

### 2.6. Next-Generation Sequencing

The amount of amplifiable DNA (ng) was calculated according to the Archer PreSeq DNA Calculator Assay Protocol (Archer DX, Boulder, CO, USA). After the fragmentation of the genomic DNA, libraries were created by the Archer VariantPlex Solid Tumor Kit (Archer DX, Boulder, CO, USA). The KAPA Universal Library Quantification Kit (Kapa Biosystems, Roche, Basel, Switzerland) was used for the final quantification of the libraries.

The MiSeq System (MiSeq Reagent kit v3 600 cycles, Illumina, San Diego, CA, USA) was used for sequencing. The libraries (final concentration of 4 nM, pooled by equal molarity) were denatured by adding 0.2 nM NaOH and diluted to 40 pM with hybridization buffer from Illumina (San Diego, CA, USA). The final loading concentration was 8 pM libraries and 1% PhiX. Sequencing was conducted according to the MiSeq instruction manual. Captured libraries were sequenced in a multiplexed fashion with a paired-end run to obtain 2 ×150 bp reads with at least 250× depth of coverage. The trimmed fastq files were generated using MiSeq reporter (Illumina, San Diego, CA, USA).

Raw sequence data were analyzed with Archer analysis software (version 6.2.; Archer DX, Boulder, CO, USA) for the presence of single-nucleotide variants (SNVs) as well as insertions and deletions (indels). For the alignment, the human reference genome GRCh37 (equivalent UCSC version hg19) was built. Molecular barcode (MBC) adapters were used to count unique molecules and characterize sequencer noise, revealing mutations below standard NGS-based detection thresholds. The sequence quality for each sample was assessed and the cutoff was set to 5% variant allele frequency (VAF). VAF is the percentage of sequence reads observed matching a specific DNA variant divided by the overall coverage at that locus. Large insertion/deletion (>50 bp) and complex structural changes could not be captured by the method. The results were described using the latest version of the Human Genome Variation Society nomenclature for either the nucleotide or protein level. Individual gene variants were cross-checked in the COSMIC (Catalogue of Somatic Mutations in Cancer) and ClinVar databases for clinical relevance. We used gnomAD v.2.1.1 population database to compare the significance of each gene alteration, which is included in our Archer NGS analysis system.

## 3. Results

### 3.1. Clinical Presentations

The clinical features of the MCC patients are shown in Table 1. The average age of the nine MCC patients was 77.8 (range: 63–89). The gender distribution was six male and three female. The localization of the tumor was more frequent in the sun-exposed regions than in other areas (e.g., gluteus). The average tumor size was 2.4 cm (range: 1–6 cm) and the mean tumor depth was 1.7 cm (range: 0.4–6.7 cm). The oncological treatments included excision, chemotherapy, and/or radiotherapy.

### 3.2. Histological Features Including Immunohistochemistry

**Figure 1:**
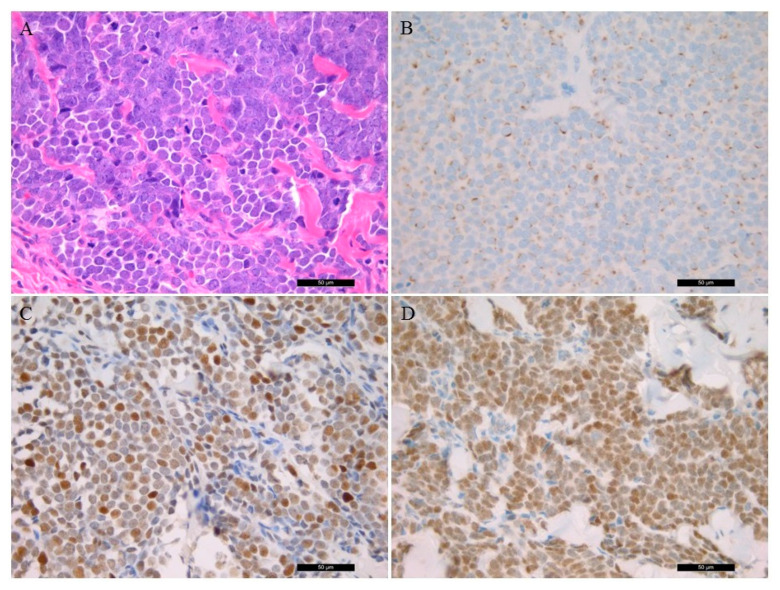
Histological and immunostaining of Merkel Cell Carcinoma (40×). (**A**) Conventional histological hematoxylin and eosin-stained characteristics of the tumor sample; (**B**) immunohistochemistry of CK20 protein; (**C**) p53 immunostaining; (**D**); Merkel cell polyomavirus large T antigen-positive tumor sample.

The tumor cells of MCC show uniform, small, blue, round cells with high nuclear/ cytoplasmic ratio with granular chromatin and numerous nucleoli (Figure 1A). Histopathological characteristics of the patient samples are presented in Table 2. Cell proliferation ratio was determined above 50%. CK20 (usually dot-like) (Figure 1B), synaptophysin, and CHGA positivity were found, while no TTF-1 expression was detected in all cases. Lymphovascular invasion was present in two cases. The p53 IHC was positive in six samples (Figure 1C). Vimentin and S100 protein positivity was detected in all melanoma samples.

### 3.3. Merkel Cell Polyomavirus Detection

MCPyV was detected with two methods. Five MCC samples were positive for the MCPyV antibody (Figure 1D). PCR amplification of the viral large T protein LT3 (amplification product size 308 bp) was positive in six cases. Additionally, in one case (case 3), only the viral capsid protein VP1 (351 bp) was positive, as well. The LT1 (439 bp) PCR was negative in all cases. Altogether, seven MCPyV-positive MCC samples were detected using PCR (7/9, 77.8% vs. 5/9, 55.6% MCPyV IHC). IHC staining had limited sensitivity in case 8 because the viral LT genome sequence was detected by PCR. In case 3 the virus LT antigene IHC did not detect positivity, aiming at viral capsid protein being present when using PCR amplification.

**Figure 2:**
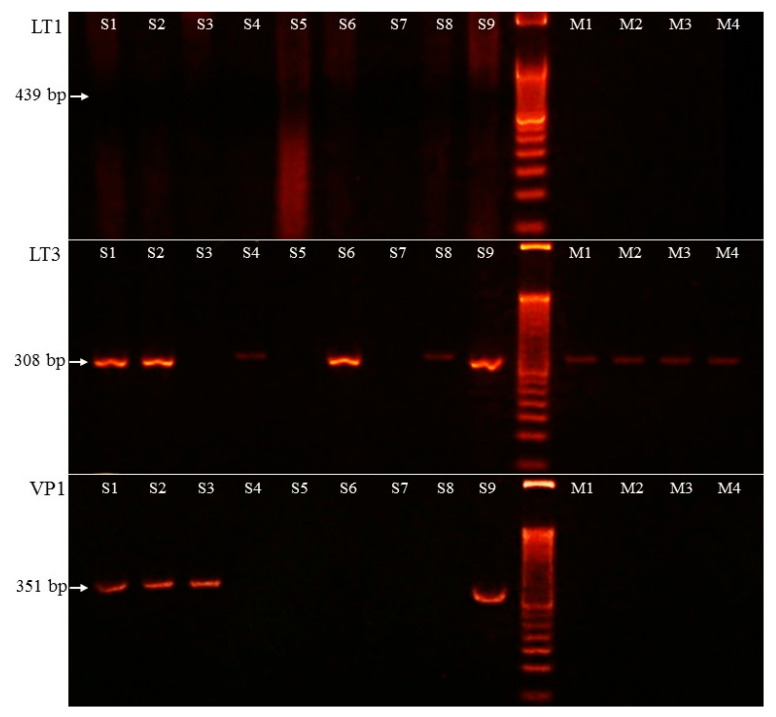
PCR amplification results of Merkel cell polyomavirus LT1 (large T1), LT3 (large T3), and VP1 (viral capsid protein 1) sequence. S1–S9: Merkel cell carcinoma samples, M1–M4: melanoma samples. PCR amplification product sizes: 439 bp (LT1), 308 bp (LT3), and 351 bp (VP1).

Two types of agarose electrophoresis lane intensity were detected and high virus copy number samples were characterized with a robust band (samples 1, 2, 6, and 9), while samples 4 and 8 showed weak stripe and, consequently, low virus copy number. The MCPyV LT3 and VP1 PCR results are presented in Figure 2.

### 3.4. Merkel Cell Polyomavirus Amplification in Melanoma Samples

PCR amplification of LT1, LT3, and VP1 virus genes was carried out on 60 melanoma DNA samples. LT3 was present in four melanoma samples with a low copy number (6.7%), opposite to LT1 and VP1, where no PCR product was amplified.

The melanoma samples were analyzed for *BRAF* mutation status using StripAssay. Thirty *BRAF* mutations were identified. In 22 cases *BRAF* c.1799T>A; p.(Val600Glu) (36.7%) and in seven samples (11.7%) the c.1798_1799GT>AA; p.(Val600Lys) mutation was detected, while in one case (1.7%) the c.1799_1780TG>AA; p.(Val600Glu) aberration was present. LT3 amplification (4/30, 13.3%) was detected only in the *BRAF*+ group with Val600Lys amino acid change. The amplification result of the four MCPyV-positive melanoma cases is presented in Figure 2.

### 3.5. NGS-Based Mutation Profiling

The 67 genes’ solid tumor panel analysis identified a series of alterations in the affected genes. The NGS analysis results are summarized in Table 3.

A *RB1* gene variant was identified in case 1 (c.2033A>T; p.(His678Leu), VAF: 48%), while *TP53* aberration was detected in case 3 (c.832C>T; p.(Pro278Ser), VAF: 92.4) and in case 9 (c.-28-4G>A, VAF: 5.5%) correlation with p53 IHC positivity was detected. Additional pathogenic variants were determined in case 3 (*FBXW7*, c.1031C>T; p.(Ser344Phe), VAF: 89.3%), in case 4 (*CTNNB1*, c.59C>T; p.(Ala20Val), VAF: 9%), and in case 6 (*HNF1A*, c.864del; p.(Pro291GlnfsTer51), VAF: 7.5%), and a benign *STK11* (c.1062C>G; p.(Phe354Leu), VAF: 54.55%) variant was detected in case 2, a *CDKN2A* (c.442G>A; p.(Ala148Thr), VAF: 50%) variant was detected in case 5, and a *JAK3* (c.2164G>A, p.(Val722Ile), VAF: 42.5%) variant was detected in case 8 was detected as well. In case 6 an *ABL1* (c.740A>G; p.(Lys247Arg), VAF: 42.1%) and a *FOXL2* (c.536C>G; p.(Ala179Gly), VAF: 65.3%) neutral aberration was identified. In MCPyV-negative case 7 no mutation was found.

Retrospectively, three MCPyV and *BRAF*-positive melanoma samples were analyzed using NGS (the fourth sample was not enough for NGS). Besides *BRAF* mutations (VAF: 40, 22.2, and 15.4%, respectively), the following aberrations were found: *PIK3CA* c.1031T>C, p.(Val344Ala, 13.4%) and *STK11* c.1211C>T, and p.(Ser404Phe, 35.1%) in M1 melanoma case, *CDKN2A* c.169dup; p.(Ala57GlyfsTer63, 20.6%) and *SMAD4* c.122A>G, p.(Glu41Gly, 5.7%) in M2; and, finally, *APC* c.3949G>C; p.(Glu1317Gln, 50%) in M3 sample (Table 3).

**Figure 3:**
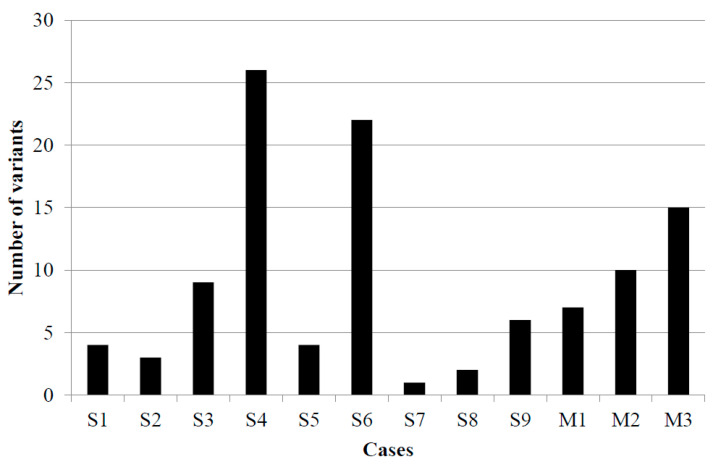
Variant tumor burden in nine Merkel cell carcinoma and three malignant melanoma cases. S1–S9: Merkel cell carcinoma samples, M1–M3: melanoma samples.

Variant tumor burden (VTB) was defined with the number of gene variants above 2% VAF (Figure 3). The largest VTB was in cases 4 and 6, while the smallest was in cases 7 and 8. Besides the MCPyV-associated case 7, in the other virus-negative case, case 5, low VTB was determined. In virus-associated melanoma cases, the average tumor burden was detected.

## 4. Discussion

In our study, we compared two methods to detect MCPyV in MCC and melanoma samples. The MCPyV monoclonal antibody, targeting the large T-antigen (clone CM2B4), effectiveness ranged from 39% to 90% [6]. The sensitivity of IHC is also related to the preanalytic varying parameters such as tissue fixation as well as viral copy numbers in the tumor cells [16]. Studies comparing PCR to IHC detection of MCPyV usually demonstrate proper concordance. Based on our results, together with other studies, we found PCR to be more sensitive than IHC [17,18,19,20]. Furthermore, we used only PCR to identify MCPyV-associated melanoma samples.

MCPyV-positive tumors have favored overall survival compared to MCPyV-negative MCCs, which also showed aggressive molecular mechanisms [8]. After viral integration, MCPyV stimulates host cell gene mutations and consequently dysregulated cell proliferation. Advances in NGS techniques have enabled the identification of these mutational landscapes that clarify the significant differences between virus-positive and virus-negative cases. As in melanoma and other skin cancers that are associated with UV radiation, these mutations (namely C-to-T pyrimidine dimers) suggest that, at least in MCV-negative tumors, the cell is unable to effectively repair UV-induced damage and subsequently accumulates further mutations.

Studies of molecular profiling on MCCs are scant and even controversial. Increased expression of the KIT receptor tyrosine kinase, in primary MCCs, demonstrated a shorter survival compared to patients with reduced levels of KIT in the malignant cells, although activating mutations in *KIT* have not been identified in MCCs [9,21]. Other genetic aberrations were also described, such as activating *PIK3CA* mutations [10], expression of the Hedgehog signaling cascade [11], aberrations of growth factors, and cell proliferation regulation [22,23]. The large T antigen regulates the life cycle of the virus and the cell cycle of the host cells. The latter is caused by interaction with the tumor suppressor gene *RB1* and *TP53* genes. The small T antigen is capable of stimulating cell proliferation through activation of several signal transduction pathways [24]. Additionally, in MCC tumor cells, small T antigen binds SCF(Fbw7) protein, thereby stabilizing large T antigen, which is a substrate for this E3 ubiquitin ligase [25]. In our study, we identified genetic variants that affected the abovementioned *RB1*, *TP53*, and *FBXW7* genes only in virus-positive MCC samples. Additional pathogenic variants were identified in *CTNNB1* and *HNF1A* genes. In one study, the *HNF1A* gene mutation involvement of the virus-positive MCC pathogenesis was identified as well and, similar to our investigation, *FGFR1*, *ABL1*, and *JAK3* variants were also demonstrated [26]. Aberrations involving *EGFR*, *FOXL2,* and *STK11* genes in MCPyV-positive MCCs had not been identified earlier.

In the literature, significant differences in the mutational burden that exists between MCPyV-positive and -negative cases were characterized, an order of magnitude higher for somatic single nucleotide variants per exome in virus-negative tumors compared to virus-positive cases [13]. Another study compared the molecular genetics of MCPyV-positive and -negative tumors and identified that MCPyV-positive MCCs harbor relatively few mutations and do not display a definitive UV-signature, verifying the oncogenic role of T-antigens as dominant drivers for these tumors [27]. One large genomics study in MCC characterized the molecular landscape of immune checkpoint inhibitor response. MCPyV sequences were detected only in low tumor burden cases. The response rate was 50% in high VTB and 41% in low VTB and MCPyV-positive tumors [28]. On the contrary, due to the limited number of MCPyV-negative cases tested, VTB was higher in MCPyV-positive samples in our study.

We found four *BRAF*-mutant MCPyV-associated melanoma cases in our study subjects, which was discordant with earlier findings, where no virus-positive melanoma cases were found and, thus, the mutation status was not analyzed [29]. In small-cell lung cancer, cancer sharing differentiation markers with MCC and MCPyV was detected in 39% [30]. Additionally, MCPyV was present in Kaposi sarcoma [31], non-melanoma skin cancers [32,33], and other cancers [34].

Several studies dealt with genetic aberrations in melanoma using NGS [35,36,37], and, according to them, the most affected genes are *BRAF*, *NRAS*, and *KIT*. In our study, in the three virus-associated cases, besides *BRAF* mutation, *PIK3CA*, *CDKN2A*, and *APC* pathogenic variants were identified. Considering the genetic similarity between virus-associated MCC and MCPyV-positive melanoma samples, no significant differentiation was observed in the tumor burden, but the affected genes were different, owing to the randomly confused signal transduction pathways by the virus.

In conclusion, MCC is an aggressive cutaneous tumor that, although rare, is increasing in incidence. We used different methodologies to analyze the samples that could provide important information for potential therapeutic options and diagnostic approaches in the future. In MCPyV-positive MCC, *RB1*, *TP53*, *FBXW7*, *CTNNB1*, and *HNF1A* pathogenic variants were identified, while in virus-negative cases only benign variants were found. In contrast to studies found in the literature, a higher tumor burden was detected in virus-associated MCC compared to MCPyV-negative cases. No association was identified between virus infection and tumor burden in melanoma samples. We concluded that analyzing the key morphologic and immunohistological features of MCC is critical to avoid confusion with other cutaneous malignancies. Molecular genetic investigations such as NGS enable molecular stratification, which may have future clinical impact.

## Figures and Tables

**Table 1:** Oncological characteristics of the Merkel cell carcinoma patients.

Patient	Gender	Age (years)	Location	Size (cm)	Depth (cm)	Treatment	Metastasis	Survival (Month)
1	M	89	left knee	4	1	excision and chemotherapy	n.a.	7
2	M	80	right face	1.5	0.9	excision	lost follow-up	
3	M	88	right chest	2.2	0.5	excision	lost follow-up	
4	F	68	left shoulder	1.1	0.7	neoadjuvant chemotherapy	axillary lymph node	24
5	M	83	right thigh	2.4	2.2	excision	lost follow-up	
6	M	76	right 3rd finger	1	6.7	excision	n.a.	9
7	F	85	right face	1.4	1.6	radiotherapy	lost follow-up	
8	F	63	right gluteus	2	0.4	radiotherapy	n.a.	6
9	M	68	right forearm	6	1	excision	lymp node	3

**Table 2:** Immunohistopathological and PCR features of the MCC patients.

Cases	Lymphocyte Infiltration	Ki-67 (%)	Cytokeratin 20	Synaptophysin	Chromogranin A	Thyroid Transcription Factor 1	p53	MCPyV IHC	LT1 PCR	LT3 PCR	VP1 PCR
1	low	60	+	+	+	-	diffuse +	+	-	+	+
2	low	60	+	+	+	-	patchy +	+	-	+	+
3	low	50	+	+	-	-	diffuse +	-	-	-	+
4	low	60	dot-like	marked	marked	-	-	diffuse +	-	+	-
5	low	80	dot-like	marked	focal	-	weakly +	-	-	-	-
6	moderate	70	dot-like	marked	marked	-	-	patchy +	-	+	-
7	low	60	dot-like	dot-like	dot-like	-	-	-	-	-	-
8	moderate	70	dot-like	+	patchy +	-	diffuse +	-	-	+	-
9	low	60	dot-like	dot-like	dot like	-	patchy +	diffuse +	-	+	+

**Table 3:** Molecular genetic NGS findings of MCC and melanoma samples. The clinical significance of gene variants was checked in the COSMIC database. The ID of nucleotide and amino acid changes were included in the table. S1–S9: Merkel cell carcinoma samples, M1–M3: melanoma samples. The PCR results of MCPyV status are presented in parentheses.

Scheme	Gene Symbol	Gene Name	Nucleotide Change	Amino Acid Change	Variant Allele Frequency (%)	Clinical Sgnificance	COSMIC ID
S1 (+)	*RB1*	retinoblastoma 1	c.2033A>T	p.His678Leu	48	no data available	-
S2 (+)	*STK11*	serine/threonine kinase 11	c.1062C>G	p.Phe354Leu	54.55	neutral	COSM21360
S3 (+)	*EGFR*	epidermal growth factor receptor	c.2137G>A	p.Glu713Lys	30.25	no data available	-
	*FBXW7*	F-box/WD repeat-containing protein 7	c.1031C>T	p.Ser344Phe	89.3		COSM1177864
	*TP53*	tumor protein P53	c.832C>T	p.Pro278Ser	92.4	pathogenic	COSM10939
S4 (+)	*CTNNB1*	catenin beta-1	c.59C>T	p.Ala20Val	9	pathogenic	COSM5702
S5 (-)	*CDKN2A*	cyclin-dependent kinase inhibitor 2A	c.442G>A	p.Ala148Thr	50	neutral	COSM3774362
	*FGFR1*	fibroblast growth factor receptor 1	c.2181-6C>T	-	48.6	no data available	-
S6 (+)	*ABL1*	tyrosine-protein kinase ABL1	c.740A>G	p.Lys247Arg	42.1	benign	-
	*FOXL2*	forkhead box protein L2	c.536C>G	p.Ala179Gly	65.3	benign	COSM4600643
	*HNF1A*	hepatocyte nuclear factor 1 A	c.864del	p.Pro291GlnfsTer51	7.5	pathogenic	COSM935974
S7 (-)	no mutation detected						
S8 (+)	*JAK3*	Janus kinase 3	c.2164G>A	p.Val722Ile	42.5	neutral	COSM34213
S9 (+)	*TP53*	tumor protein P53	c.-28-4G>A	-	4.5	no data available	-
M1 (+)	*BRAF*	serine/threonine kinase BRAF	c.1799_1800delinsAA	p.Val600Glu	40	pathogenic	COSM475
	*PIK3CA*	phosphatidylinositol bisphosphate 3-kinase	c.1031T>C	p.Val344Ala	13.4	pathogenic	COSM86951
	*STK11*	serine/threonine kinase 11	c.1211C>T	p.Ser404Phe	35.1	no data available	-
M2 (+)	*BRAF*	serine/threonine kinase BRAF	c.1799T>A	p.Val600Glu	22.2	pathogenic	COSM476
	*CDKN2A*	cyclin-dependent kinase inhibitor 2A	c.169dup	p.Ala57GlyfsTer63	20.6	pathogenic	COSM110662
	*SMAD4*	mothers against decapentaplegic homolog 4	c.122A>G	p.Glu41Gly	5.7	no data available	-
M3 (+)	*BRAF*	serine/threonine kinase BRAF	c.1799T>A	p.Val600Glu	15.4	pathogenic	COSM476
	*APC*	adenomatous polyposis coli	c.3949G>C	p.Glu1317Gln	50	pathogenic	COSM19099

## Data Availability

The data presented in this study are available on request from the corresponding author. The data are not publicly available due to protecting the rights of patients.
